# Antibacterial Activityand Comparison of the Volatile Oils of *Tanacetum tenuisectum* (Boiss.) Podl. Obtained by Three Different Methods of Extraction

**Published:** 2017

**Authors:** Shiva Masoudi, Jila Abbassi

**Affiliations:** *Department of Chemistry, Islamic Azad University, Central Tehran Branch, Tehran, Iran.*

**Keywords:** *Tanacetumtenuisectum*, Compositae, Essential oil, Hydrodistillation, Steam distillation, Microwave extraction, Antibacterial activity

## Abstract

The essential oils obtained by hydrodistillation (HD), steam distillation (SD) and solvent free microwave extraction (SFME) from the stems and flowers of *Tanacetumtenuisectum* (Boiss.) Podl., which is endemic to Iran, were analyzed by combination of GC and GC/MS.

Camphor (26.91 %, 27.23% and 25.52%), borneol (12.61%,11.48% and 7.62%) and 1,8-cineole (7.93%, 13.23% and 11.26%) were the main constituents of the HD,SD and SFME oils of *Tanacetumtenuisectum* respectively.

All three oils were rich in regard to monoterpenes and small percentage of sesquiterpenes and non terpenoid compounds.

Antibacterial activity of the essential oil of the plant was determined against six Gram positive and Gram negative bacteria. The results showed that this oil was active against all of the tested bacteria.

## Introduction

The genus *Tanacetum*, which is an important member of the Compositaefamily, is widespread in Europe and Western Asia and consists about 150-200 species.The flora of Iran comprises 26 species of *Tanacetum*ofwhich 12 are endemic (1, 2).

Some members of this genus have traditionally been used as a spicy additive for food, in cosmetics and as herbal remedies due to their biologically active compounds (3). Especially *Tanacetumparthenium*hasbeenused sinceancienttimes for a variety of medicine proposes, and recently has gained considerableprominence due to its ability of alleviating the symptoms of migraine, athritisand psoriasis, and to inhibition of blood platelet aggregation (4).

According to recent studies, essential oils and extracts of members of the genus *Tanacetum*exhibit anti-inflammatory (5-7) anticancer (8) antibacterial (9, 10) antiviral (11) antifungal (12) insecticidal (13) and antiprotozoal effects (14, 15).

This genus is also found to contain sesquiterpene lactones (16, 17) a large group of molecules with several biological activities (18, 21).

Previous chemical investigations on different Iranian species of*Tanacetum* have shown that they possess sesquiterpene lactones (22, 23) and essential oils (24-32).

As part of our ongoing work on the chemical analysis of volatile obtained from wild plants of Iran, we report the composition of the volatile oils of *Tanacetumtenuisectum* obtained by hydrodistillation, steam distillation and solvent free microwave extraction with its antibacterial activity.

## Experimental

*Plant material*

The stems and flowers of *Tanacetumtenuisectum (*syn*. Pyrethrum tenuisectum*Boiss., *Chrysanthemum gaubae*Bornm. and *Chrysanthemum tenuisectum* (Boiss.) Parsa), which is endemic to Iran, was collected from Rameh area, Garmsar, Province of Semnan, Iran in July 2013, during the flowering stage.

Voucher specimens have been deposited at the Herbarium of the Research Institute of Forests and Rangelands (TARI), Tehran, Iran.

*Isolation of the essential oils*

*Distillation*

Air-dried ground stems and flowers of the plant were separately subjected

tohydrodistillation and steam distillation using a Clevenger type apparatus for 3h. The obtained essential oils were dried over anhydrous sodium sulfate and after filtration, stored at 4ºC until tested and analyzed. The yield was found to be 0.2% and 0.3% (w/w), respectively.

*Solvent Free microwave extraction*

SFME extractionwas performed in a Milestone ETHOS 1600 batch reactor, which is a multimode microwave reactor operating at 2455 MHZ with a maximum delivered power of 1000 W, variable in 10 W increments. The dimensions of the PTFE-coated cavity are 35×35×35 cm. During the experiment time, temperature, pressure, and power were controlled using the ^"^easy-WAVE^"^ software package. Temperature was monitored with the aid of a shielded thermocouple (ATC-300) inserted directly in to the sample container. 

Ina typical SFMEprocedure, 250 g of dry stems and flowers of *T.tenuisectum* were moistened prior to extraction by soaking in water for 1 h, then draining off the excess water.This step is essential to give thestems and flowers the initial moisture. Moistened stems and flowers were next placed in a reactor without any added solvent or water. The essential oil is collected, dried with anhydrous sodium sulfate and stored at 0ºC until used.

*Gas chromatography*

GC analysis was performed on Schimadzu15 A gas chromatograph equipped with a split/splitless injector (25ºC) and a flame ionization detector (250ºC). Nitrogen was used as carrier gas (1 mL/min) and the capillary column used was DB-5 (50m×0.2mm, film thickness 0.32*µ*m).The column temperature was kept at 60ºC for 3 min and then heated to 220ºC witha 5ºC/ min rate and kept constant at 220ºC for 5 min. Relative percentage amount were calculated from peakarea usinga SchimadzuC-R4 Achromatopac without the use of correction factors.

*Gas chromatography - mass spectrometry*

Analysis was performed using a Hewlett- Packard 5973 with a HP-5MS column (30m×0.25 mm, film thickness 0.25 *µ*m). The column temperature waskept at 60ºC for 3 min and programmed to 220ºC at a rate of 5ºC/min and kept constant at 220ºC/min for 5 min.The flow rate of Helium as carrier gas with (1 mL/min).

Mass spectrometry wastaken at 70 eV. The retention indices for all the components were determined according to the Van Den Door method, using n- alkanes as standards (33).

The compounds were identified by (RRI, DB5) with those reported in the literature and by comparison of their mass spectra with the Wiley library or with the published mass spectra (34, 35).

*Antibacterial assay*

The antibacterial activity of the essential oil from the aerial parts of *Tanacetumtenuisectum* was evaluated by disc diffusion method using Mueller- Hinton Agar (36). The antibacterial activity of the essential oil of the plant was tested against three Gram- positive and three Gram-negative bacteria. 

The Gram-positive bacteria included *Staphylococcus aureus*ATCC 25923, *Bacillus subtilis* ATCC 9372, and *Bacillus creus* ATCC 6633, and the Gram-negative bacteriaincluded *Klebsiellapneumoniae* ATCC 27736, *Entobacteraerogenes* ATCC 49469, and *Escherichia coil* ATCC 25922. The bacteria were obtained from the Iranian Research Organization of Science and Technology. 

A serial dilution of the oil was prepared in Mueller-Hinton Broth forbacteria. The oil was diluted by the water and ethanol solvents. The solvents, at an appropriate concentration were also used as a negative centrol. The standardized suspension of bacteria was incubated in to each tube. The tubes were incubated at37ºCfor 24h.Thelowestoil concentration, where there was no visible growth, was the Minimum Inhibitory Concentration (MIC) when compared to control.

To determine the Minimum Bactericidal Concentration (MBC), for each set of test tubes in the MIC determination, a loopful of broth was collected from those tubes which did not show any growth and incubated on Muller- Hinton Agar by streaking. Plates incubated with bacterial were then incubated at 37ºC for 24 h. After incubation, the concentration at which no visible growth was seen was noted as MBC for bacteria. All the experiments were carried out in triplicate and mean calculated.

## Results and Discussion

The identified volatile components and their peak area percentages of the stems and flowers of *Tanacetumtenuisectum*obtained by hydrodistilliation, steam distillation and solvent free microwave extraction are given in Table 1. The components are listed in order of their elution on the DB-5 column.

As it is shown from the Table 1, about 96.87% (34 components) of the hydrodistilled oil, 91.32% (46 constituents) of the steam distilled oil and 95.1% (44 components) of the solvent free microwave extraction oil of *T.tenuisectum*were identified. The main components in three oils were camphor (26.91%, 27.23% and 25.52%), borneol (12.61%, 11.48% and 7.62%) and 1,8- cineole (7.93%, 13.23% and 11.26%), respectively. Other notable constituents were in hydrodistilled oil hexadecanoic acid (7.30%), carotol (6.58%) and γ-eudesmol (5.75%); in steam distilled oil camphene (5.44%), hinesol (4.35%) and (E)-sequilavandulol (4.24%) and in solvent free microwave extraction oil hinesol (6.86%), hexadecanoic acid (6.55%) and (E) - sequilavandulol (4.89%).

**Table 1:** Comparative chemical composition (%) of *Tanacetumtenuisectum* oil obtained by HD, SD and SFME.

**No.**	**Compounds** a	**RI** b	**HD(%)**	**SD(%)**	**SFME(%)**
1	Hexanal	800	-	0.24	-
2	Tricyclene	924	-	0.37	-
3	α-Thujene	928	-	0.19	t
4	α- Pinene	935	-	1.51	1.00
5	Camphene	951	0.70	5.44	3.92
6	*β*- Pinene	981	-	1.04	0.68
7	Mesitylene	994	-	0.33	-
8	*p*- Cymene	1024	t	1.84	1.17
9	1,8-Cineole	1033	7.93	13.23	11.26
10	γ-Terpinene	1062	-	0.42	t
11	Linalool	1096	0.73	0.62	t
12	Camphor	1141	26.91	27.23	25.52
13	*cis*-Chrysanthenol	1160	0.82	-	1.09
14	Pinocarvone	1162	-	0.32	-
15	Borneol	1165	12.61	11.48	7.62
16	Terpin-4-ol	1177	1.14	0.99	0.59
17	Naphthalene	1183	-	0.25	-
18	α-Terpineol	1188	1.20	0.84	0.58
19	Myrtenal	1191	-	0.22	-
20	Myrtenol	1193	-	0.24	-
21	*trans*-Carveol	1217	0.64	0.78	0.54
22	*cis*-Chrysanthenyl acetate	1260	t	0.23	t
23	Bornyl acetate	1285	1.66	1.40	1.12
24	2- methyl Naphthalene	1292	-	1.00	0.86
25	1- methyl Naphthalene	1306	-	0.63	t
26	α-Terpinyl acetate	1350	-	0.3	-
**No.**	**Compounds** ^a^	**RI** ^b^	**HD(%)**	**SD(%)**	**SFME(%)**
27	Decanoic acid	1352	2.03	-	0.19
28	α-Copaene	1376	-	0.27	-
29	2,6-dimethyl Naphthalene	1379	-	-	0.57
30	(E)-*β*-Damascenone	1380	-	t	-
31	1,7- dimethyl Naphthalene	1397	-	-	0.44
32	2,7- dimethyl Naphthalene	1400	-	0.54	-
33	1,3- dimethyl Naphthalene	1415	-	0.46	-
34	*β*-Caryophyllene	1418	-	-	0.73
35	Neryl acetone	1431	0.80	-	0.88
36	isobutyl-n-Butyrate	1471	1.12	-	-
37	Pentadecane	1500	-	0.17	-
38	*β*-Bisabolene	1509	-	-	0.56
39	Elemol	1546	-	-	0.41
40	(E)- Nerolidol	1564	1.07	0.36	1.37
41	(z)-dehydroApofarnesol	1568	0.70	-	0.77
42	Spathulenol	1574	2.75	1.11	1.37
43	Caryophyllene oxide	1581	3.43	1.40	2.01
44	Carotol	1594	6.58	-	-
45	Tetradecanal	1610	1.30	-	1.23
46	(E)- isoEugenol acetate	1611	-	0.39	-
47	γ-Eudesmol	1630	5.75	-	-
48	(E)- Sesquilavandulol	1632	-	4.24	4.89
49	*β*- Caryophylla-4(12),8(13)dien-5-ol	1636	1.87	-	0.87
50	Hinesol	1638	-	4.35	6.86
51	*β*-Eudesmol	1646	-	1.58	2.33
52	α-Eudesmol	1652	1.95	-	-
53	α-Cadinol	1653	-	-	1.41
54	1(5),3-Aromadenedriene	1660	-	-	0.51
55	14- hydroxy-9-epi-*β*- Caryophyllene	1664	0.97	0.27	-
**No.**	**Compound**s^a^	**RI** ^b^	**HD(%)**	**SD(%)**	**SFME(%)**
56	Khusinol	1674	-	0.31	-
57	(z)-Nerolidol acetate	1675	0.79	-	-
58	1,6-dimethyl-4-(1-methylethyl) Naphtalene	1685	-	0.30	0.98
59	Germacrone	1693	0.86	-	-
60	2-Pentadecanone	1696	-	-	0.61
61	(E)- Neroliol acetate	1712	0.55	-	-
62	(E,E)- Farnesol	1722	-	-	0.38
63	Tetradecanoic acid	1771	0.61	0.50	1.18
64	Octadecane	1800	t	0.21	-
65	Hexadecanal	1806	-	-	0.64
66	6,10,14-trimethyl-2-Pentadecane	1872	0.80	0.44	0.88
67	Nonadecane	1900	0.67	0.18	-
68	Hexadecanoic acid	1973	7.30	2.76	6.55
69	Eicosane	2000	0.63	-	-
70	Henicosane	2100	-	0.34	0.53
	Monoterpene hydrocarbons		0.70	10.81	6.77
	Oxygenated monoterpenes		54.44	57.88	49.20
	Sesquiterpene hydrocarbons		-	0.27	1.29
	Oxygenated sesquiterpenes		27.27	13.62	23.18
	Other compounds		14.46	8.74	14.66
	Total		96.87	91.32	95.1

t= trace ( < 0.1% ) .

Note: a Compounds listed in order of elution from HP-5 MS column;

b Retention indices to C_8_ - C_24 _ n-alkanes on HP-5 MS column;

According to these results, the composition of the three oils show significant similarity for the concentration of the main components. All three oils were rich in regard tomonoterpenes (55.14%, 68.69%and 55.97%, respectively), while the sesquiterpenes fraction was (27.27%, 13.89% and 24.47%, respectively). The nonterpenoid fraction was relatively small, representing 14.46%, 8.74%, and 14.66%, respectively.

In our pervious investigations on *Tanacetum* genus we have identified essential oil compositions of *T. balsamitha*,* T. polycephalum*,*T.khorassanicum*,*T. paradoxum*,*T. tabrisianum*, *T. elburensis*and *T.persicum*(24-28). The dominant compound in *T. balsamitha* was carvone (68.0%) (24). Camphor (18.2% and 13.9%) and 1,8- cineole (17.0% and 18.6%) were found to be the major components of the oil of *T. polycephalum* and *T. lingulatum*, respectively (25, 29).

The major constituents of the aerial partsof *T. khorassanicum* were (E)-myroxide (19.8%), camphor (16.4%), isopulegon (13.4%) and 1,8-cineole (11.4%) (26).

Water distilled oil obtained from the aerial parts of *T. paradoxum* and *T.tabrisianum* have been the subject of our previous studies. The major components were camphor (23.8%), lavandulyl acetate (19.1%), lavandulol (15.9%) and 1,8- cineole (13.2%) in the former oil, caryophyllene oxide (12.0%)and spathulenol (10.3%) in the latter (27). Water- distilled oils from the aerial parts of *T. elburensis* and *T. persicum*growing wild in Iran were investigated .

The main constituents of the oil of *T. elburensis* were menthylisovalerate (20.0%) and 1,8 cineole (16.6%). The oil of *T. persicum* was characterized by higher amounts of borneol (24.3%), menthyl acetate (17.3%), isobornyl 2- methyl butyrate (16.0%) and artedouglasia oxide D (14.3%) (28).

The oils obtained by hydrodistillation of the leaves and flowers of T.*dumosum* growing wild in Iran were investigated. The main constituents of the leaves oil were borneol (27.9%), bornylacetate (18. 4%) and 1,8- cineole (17.5%), while the main components of the flower oil were isobornyl-2-methylbutanoate(41.1%) and*trans*-linalyl oxideacetate (11.9%) (30).

*Aerial parts of T. sonbolii, endemic in Iran,contained α- cadinol (35.3%),*globulol (20.1%) and 1,8-cineole (8.6%) as major constituents (31).

Camphor (30.2%), (z)-chrysanthenylacetate (26.5%) and α- farnesene (11.1%) were found to be the major components from the root oil of *T.parthenium*from Iran. (32).

The dominant compound in the flower and stem oils of*T. chiliophyllum*, from Turkey, was camphor (17.3% and 10.4%) respectively,while root oil of the plant was characterized with hexadecanoic acid (37.5 %) (37).

The dominant compound in the oil of *T. nitens* and *T. argenteum* from Turkey was 1,8 cineole (27.57%) and α- pinene (27.86%) respectively (38).

Baser *et al*. reported the oils from the flowers of *T. zahlbruckneri* and flowers and stems of *T. tabrisianum* from Turkey.

The flower oil of *T. zahlbruckneri* was characterised by germacreneD (29.7%) andspathulenol(12.0%).1,8-Cineole (17.6% and 22.5%) and hexadecnoic acid (10.3% and 8.0%) were the major constituents of the flower and stem oil of *T. tabrisianum* respectively (39).

The flower, stem and leaf oils of *T. densum* fromTurkey,were characterized with camphor (30.9%,25.7% and 27.7%, respectively) (40).

Lavandulol (21.5%) and 1,8- cineole (15.2 %) were identified as major components in the oil of *T. gracile* from LadakhHimalaya (India) (41).

Results of the antibacterial activity of the essential oil of *Tanacetumtenuisectum*are shown in Table 2.

**Table 2:** Antibacterial activity of essential oil of *Tanacetumtenuisectum*

**Microorganisms**	**Inhibition zone(mm)**	**MIC** a	**MBC** b
*Staphyloccusaureus* (ATCC 5923)	11	625	1250
*Escherichia coil* (ATCC 25922)	13	625	1250
*Klebsiellapneumoniae* (ATCC 27736)	16	625	1250
*Entobacteraerogenes* (ATCC 49469)	16	625	625
*Bacillus subtilis* (ATCC 9372)	13	625	625
*Bacillus creus* (ATCC 6633)	16	1250	2500

MIC and MBC of compounds are indicated in*µg***/**mL.

Note: a Minimum inhibitory concentration;

b Minimum bactericidal concentration .

Anti bacterial activity was determined against six bacteria. 

The oil has shown maximum zone of inhibition against *Klebsiellapneumonia*e, *Entobaceraerogenes* and *Bacillus creus*. *Staphylococcus aureus*, *Escherichia coli*, *Klebsiellapneumoniae*, *Entobacteraerogenes* and *Bacillus subtilis* were the most sensitive bacteria to the essential oil (having MIC value 625 *µ*g/mL). 

*Entobacteraerogenes* and *Bacillus subtilis* have a minimum bactericidal concentration (MBC value 625 *µ*g/mL). 1,8- Cineole and camphor are well- known chemicals having antibacterial potentials (42, 43).

The antibacterial effects of borneol were also reported (44). As aresult of these findings, antibacterial activity of *T. tenuisectum* oil could be attributed to 1,8 cineol, camphor and borneol. The present study confirms that there is a positive correlation between the chemical content of the oils and their antibacterial activities.
